# Improving Hepatitis B Vaccination Rates among At-risk Children and Adolescents with Inflammatory Bowel Disease: Erratum

**DOI:** 10.1097/pq9.0000000000000584

**Published:** 2022-08-01

In “Improving Hepatitis B Vaccination Rates among At-risk Children and Adolescents with Inflammatory Bowel Disease,” published 23 June 2022, one of the author’s name was incorrect. The correct name is Megan McNicol, PharmD, BCACP.
